# Flavonoid Profile of the *Genista tridentata* L., a Species Used Traditionally to Treat Inflammatory Processes

**DOI:** 10.3390/molecules25040812

**Published:** 2020-02-13

**Authors:** Mark A. M. Simões, Diana C. G. A. Pinto, Bruno M. R. Neves, Artur M. S. Silva

**Affiliations:** 1 LAQV-REQUIMTE, Department of Chemistry, University of Aveiro, Campus de Santiago, 3810-193 Aveiro, Portugal; mark.simoes@outlook.com; 2 Department of Medical Sciences and Institute of Biomedicine—iBiMED, University of Aveiro, 3810-193 Aveiro, Portugal; bruno.neves@ua.pt

**Keywords:** *Genista tridentata*, UHPLC-DAD-ESI/MS^n^ profile, flavonoids, lupinifolin, mundulin, 3-methoxymundulin, anti-inflammatory activity

## Abstract

Ethnopharmacological surveys on Portuguese flora reveal that *Genista* *tridentata* L. is a shrub used in traditional medicine for the treatment of various inflammation-related health problems, although scientific support of its benefits is still necessary. In order to establish the anti-inflammatory potential of *G. tridentata* and support its traditional use, ethanolic extracts of three sections of the plant (root, stem, and leaves) were subjected to in vitro evaluation of anti-inflammatory activity using lipopolysaccharide (LPS)-stimulates macrophages as an inflammation model. Simultaneously, we also aimed to establish the extracts’ flavonoids profile. The ethanolic extracts, obtained by Soxhlet extraction, profile of the three sections confirmed their richness in flavonoids, being three prenylated flavonoids isolated and characterized in the root, including a new natural compound, the 3-methoxymundulin. The extracts from the three plant sections showed strong antioxidant activity at the cellular level and significantly inhibit the LPS-triggered NO production by downregulating *Nos2* gene transcription and consequently iNOS expression. Additionally, root and stem extracts also decreased the LPS-induced transcription of the pro-inflammatory genes *Il1b*, *Il6*, and *Ptgs2*. Thus, the results support the anti-inflammatory properties attributed to *G. tridentate* preparations. Relevantly, the roots of the shrub, plant part not used, is an unexplored source of compounds with pharmacological and nutraceutical value.

## 1. Introduction

*Genista tridentata* L. [1] (Note: *Genista tridentata* L. is the accepted name of the species and have 13 synonyms and three infraspecific taxa accepted. Among the synonyms *Pterospartum tridentatum* (L.) Willk. is the most used in the literature and also in the commercially available extracts.) is an endemic shrub of the Iberian Peninsula belonging to the Leguminosae family, locally known as carqueja or carqueija [2]. It grows up spontaneously in the north-eastern region of Portugal, showing coriaceous winged stems, alternate branches, and yellow inflorescences [3]. The flowers are usually collected during spring and used to flavor rice or roasted meat or dried for subsequent preparation of decoctions and infusions. These hot water extracts are believed in folk medicine to present hypoglycemic, anti-hypertensive, and depurative effects being also used for the treatment of numerous inflammation-related health problems [4,5]. The water infusions of the shrub aerial parts are rich in phenolic compounds and have strong in chemico antioxidant activity [6,7,8]. In vitro experiments corroborated the antioxidant properties, with human endothelial cells being protected from H_2_O_2_-induced oxidative injury, an effect attributed to isoquercitrin [9]. Flavonols, flavones, and isoflavones are highly abundant in the hidroalcoholic extracts of the plant and confer them a strong antibacterial activity against methicillin-resistant and methicillin-sensitive *Staphylococcus aureus* [10]. Finally, the polysaccharides present in the dried inflorescence of *G. tridentata* were also pointed as possible contributors to its health beneficial effects, namely through relevant anti-inflammatory activity [11].

Inflammation is subjacent to multiple health problems, and its mitigation is therefore highly desirable in the treatment of diseases such as rheumatoid arthritis, osteoarthritis, diabetes, atherosclerosis, and Alzheimer’s [12,13]. However, available anti-inflammatory drugs have multiple limitations and the development or discovery of novel and safe compounds is of great interest.

The above-mentioned works deal with the aerial parts or flowers and are responsible for the isolation and identification of six isoflavones and three flavone derivatives, from which genistein, prunetin, rutin, and isoquercitrin are due to some already established biological properties [6,7,14,15,16,17,18].

Here, we sought to chemically characterize the ethanolic extracts of the root, stem, and leaves of *G. tridentata* and to address their antioxidant and anti-inflammatory activities in vitro. Lipopolysaccharide-stimulated Raw 264.7 macrophages were used as an inflammation model and the effect of extracts on the production of NO and ROS (reactive oxygen species), as well as on the transcription of *Nos2*, *Il1b*, *Il6*, *Ptgs2*, and *Tnfa* was analyzed. Our results support the anti-inflammatory effects attributed in folk medicine to *G. tridentata* and provide evidence for the possible use of roots and leaves as a source of compounds with valuable pharmacological or nutraceutical properties.

## 2. Results and Discussion

### 2.1. Biological Activity of G. Tridentata Ethanolic Extracts

**Figure 1:**
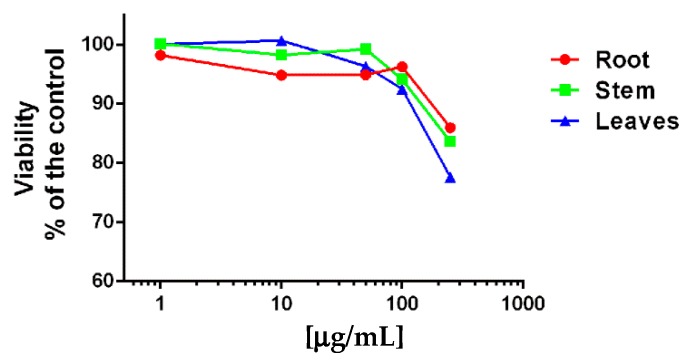
Effect of *G. tridentata* extracts on Raw 264.7 macrophages viability. Cells were exposed to different concentrations of the extracts, during 24 h and then viability was assessed by the resazurin assay. Data is presented as percentage of untreated cells (control) and represent the mean from three independent experiments.

Some of the health benefits attributed in popular medicine to *G. tridentata* infusions or decoctions are related to their anti-inflammatory potential [4,5]. However, studies directly addressing these pharmacological effects in *G. tridentata* preparations are scarce. Thus, the antioxidant and anti-inflammatory activities of ethanolic extracts of the root, stem, and leaves of *G. tridentata* was evaluated using LPS-stimulated macrophages as an in vitro inflammation model. First, the impact of the extracts on cell viability was addressed in order to select the concentration to be used in subsequent assays. In the range of concentration studied (10 to 250 mg/mL) the root extract was shown to be less cytotoxic while the extract from leaves caused the higher impact on cell viability (Figure 1). Given the data obtained, the concentration 100 μg/mL was selected, since in all extracts it resulted in no more than 5% to 10% cytotoxicity.

**Figure 2:**
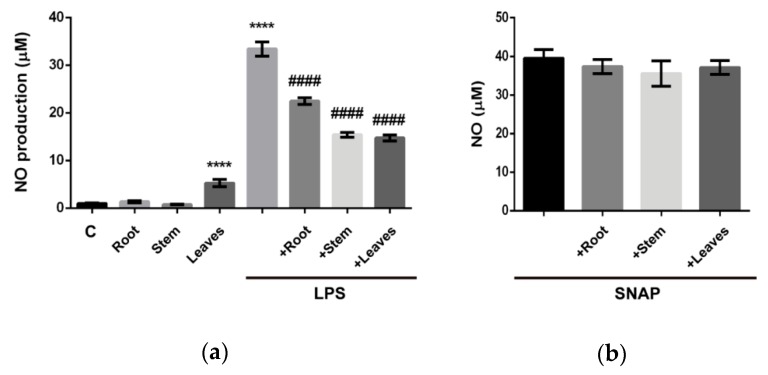
Evaluation of the capacity of *G. tridentata* extracts to modulate NO production in Raw 264.7 macrophages. (**a**) Levels of NO in supernatants from cells stimulated with extracts or with extracts and LPS. (**b**) Assessment of the potential of NO scavenging activity through the SNAP assays. Data is presented as concentration of NO and represent mean ± SD from three independent biological experiments. (**** *p* < 0.0001: control vs. LPS; #### *p* < 0.0001: LPS vs. extracts + LPS). Data is presented as percentage of control (nontreated cells/medium) and represent the mean ± SD from at least three independent experiments.

The production of NO by macrophages is critical for the resolution of infectious processes, but its excessive and/or continued production is strongly connected to the development of chronic inflammatory diseases [19]. Therefore, if extracts induce NO *per se* and if they were able to modulate its LPS-induced production was evaluated (Figure 2a). The incubation of cells with extract from roots or stems did not significantly increase NO levels while extract from leaves caused a slight increase. Looking at the modulation of LPS-triggered NO production it was noticed that all parts of the plant caused a significant decrease (Figure 2a) with this effect as not attributable to direct NO scavenging activity (Figure 2b).

**Figure 3:**
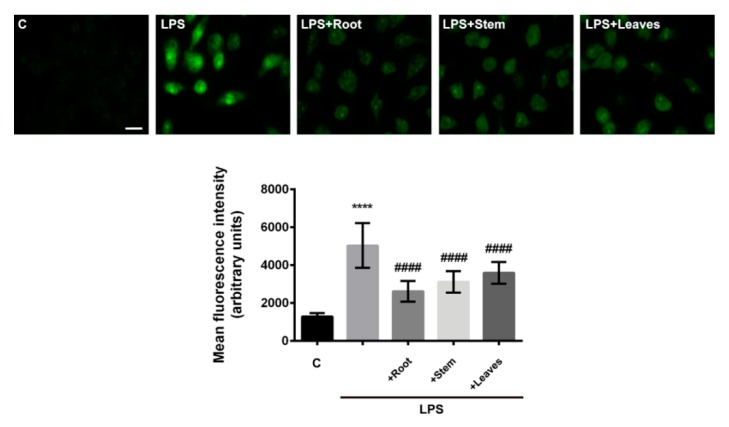
Effects of *G. tridentata* extracts on the LPS-triggered production of ROS by macrophages. Cells were cultured in the indicated conditions and the ROS production was assessed with H_2_DCFDA (green), a ROS-sensitive fluorescent probe. Hoechst (blue) was used to label the nuclei. Images representative of different fields were acquired at a magnification of 63 × (scale bar = 20 µm). Data is presented as mean fluorescence intensity ± SD from three independent experiments. (**** *p* < 0.0001: C vs. LPS; #### *p* < 0.0001: LPS vs. extracts + LPS).

The pre-treatment of macrophages with extracts also significantly reduced cellular ROS levels resultant from LPS stimulation (Figure 3). This observation directly relies on the well-established antioxidant properties of flavonoids, metabolites previously found in this species [6,7,14,20], and is in accordance with previous studies demonstrating strong *in chemico* and in vitro antioxidant activity of *G. tridentata* extracts [6,21,22]. There are evidences in the literature that at least genistein, biochanin A, and daidzein can contribute to the observed effects [23,24]. These compounds have direct ROS scavenging activity and were also shown to increase the expression of superoxide dismutase and glutathione peroxidase, key enzymes in the protection of cell from oxidative damage [25,26].

**Figure 4:**
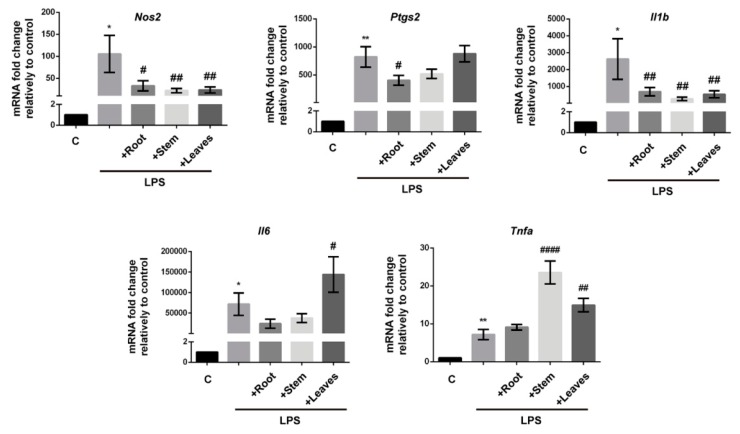
Effects of *G. tridentata* extracts over LPS-induced transcription of *Nos2*, *Ptgs2*, *Il1b*, *Il6,* and *Tnfa* genes. Cells were exposed to extracts from root, stem, and leaves and then stimulated with LPS. After 24 h the mRNA was extracted and the gene transcription was assessed by q-PCR. Data is presented as mRNA fold change relatively to untreated cells (C) and represent the mean ± SD from three independent experiments. (* *p* < 0.05; ** *p* < 0.01: C vs. LPS; # *p* < 0.05; ## *p* < 0.01; #### *p* < 0.0001: LPS vs. extracts + LPS).

**Figure 5:**
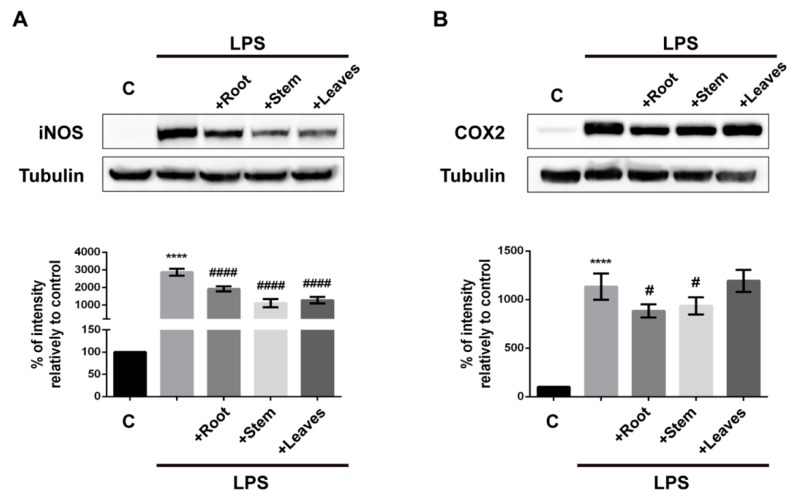
Impact of *G. tridentata* extracts on the LPS-induced expression of iNOS and COX-2. Cells were exposed to extracts from root, stem, and leaves and then stimulated with LPS. After 24 h total cell lysates were prepared and iNOS and COX-2 protein levels were assessed by Western blot. The results are expressed as percentage of optical densities relative to untreated cells (control). Equal protein loading was controlled using antibody against β-tubulin. A representative blot is shown and each value on graphs represents the mean ± SD of three independent experiments. (**** *p* < 0.0001: C vs. LPS; # *p* < 0.05, #### *p* < 0.0001: LPS vs. extracts + LPS).

In order to have a deep understanding on the anti-inflammatory potential of *G. tridentata* extracts, their capacity to modulate the LPS-triggered transcription of *Nos2* and *Ptgs2* genes was analyzed by qPCR (Figure 4) and the quantification of the respective coded proteins iNOS and COX-2 was performed by Western blot (Figure 5). The effects on mRNA levels of *Il1b*, *Il6*, and *Tnfa* were also addressed. All the extracts significantly decreased *Nos2* mRNA levels resulting in a correspondent decrease in protein expression. Therefore, the noticed decrease of LPS-triggered NO production caused by extracts is due to their capacity to limit Nos2 gene transcription, hypothetically by interfering with pro-inflammatory transcription factors such as nuclear factor-κB (NF-κB) and signal transducer and activator of transcription 1 (STAT-1). In fact, the flavonoids genistein, prunetin, biochanin A derivatives, and kampferol have been shown to inhibit NO production by decreasing the activation of NF-κB signaling pathway [15,16,27,28,29].

When analyzing the effects over *Ptgs2* transcription it was found that significant decreases are only obtained by treatment with extracts from roots (Figure 4). Determination of COX-2 protein levels, the correspondent translation product, followed the same profile, with root extracts being the most effective in limiting the LPS-triggered expression (Figure 5). Given the pivotal role of COX-2 in inflammation we can speculate that, compared to the other plant parts, preparations from *G. tridentata* roots would have superior anti-inflammatory activity as they would limit prostaglandin production. Again, this effect may be due to the presence of compounds such as genistein and daidzein. Genistein and daidzein are known to exhibit strong inhibitory activity over NF-κB by blocking the degradation of inhibitory κB-α (IκB-α) which results in decreased nuclear translocation of the NF-κB p50 subunit [17,30]. Additionally, these isoflavones were also shown to block STAT-1 phosphorylation, another transcription factor involved in the transcription of *Nos2* and *Ptgs2* [17,30].

Regarding the effects on the transcription of the other studied inflammatory mediators, the results varied according to the gene and to the plant portion from which the extract was obtained. While LPS-induced transcription of *Il1b* was significantly decreased by all extracts, *Il6* and *Tnfa* genes were either not modulated or their mRNA levels paradoxically increase (Figure 4). The referred effect is particularly evident for *Tnfa* which was induced by stem and leaf extracts. These results may be explained by the different transcription factors involved in the regulation of each gene. While *Il1b* is mainly regulated by NF-κB, a pathway known to be inhibited by several flavonoids, *Il6* and *Tnfa* genes are additionally regulated by other transcription factors. *Il6* is regulated by activator protein (AP)-1, CCAAT/enhancer binding protein (C/EBP), and cAMP response element-binding protein (CREB) [31] and *Tnfa* by nuclear factor of activated T-cells (NFAT) along with Sp1 and Ets/Elk [32]. Thus, by our results we can infer that the compounds present in extracts from stem and leaves do not inhibit these transcription factors and in contrast can activate some of them causing the observed synergism with LPS (Figure 4). This reinforces the notion that plant preparations used in folk medicine may contain numerous compounds, some with beneficial properties but others with undesirable characteristics.

### 2.2. Ethanolic Extracts UHPLC-DAD-ESI/MS^n^ Analysis

**Figure 6:**
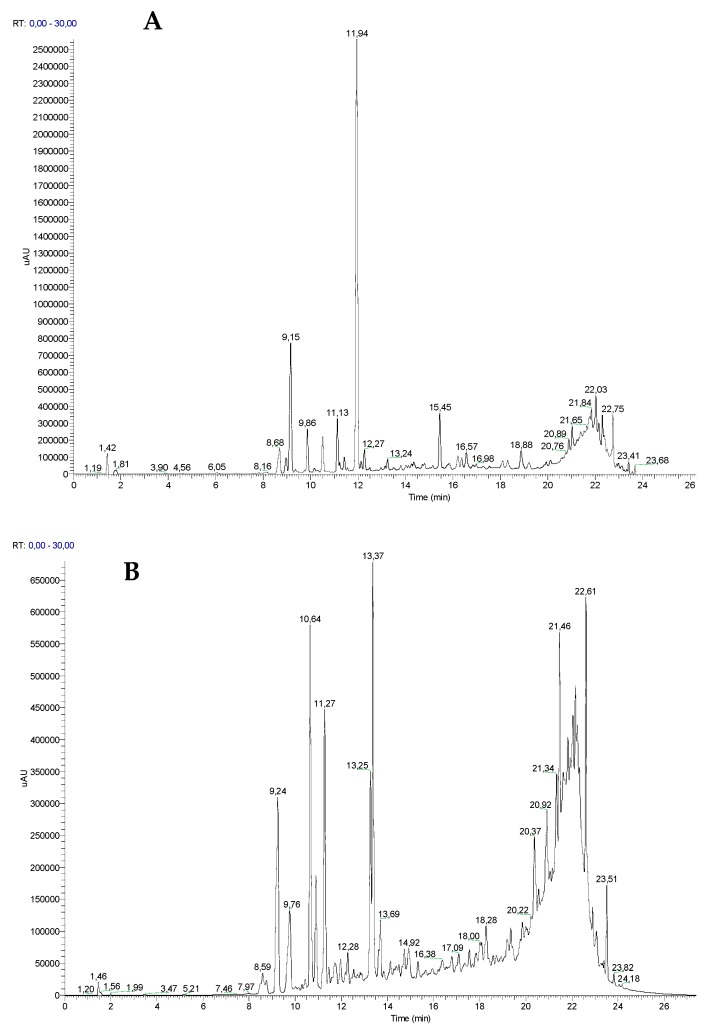

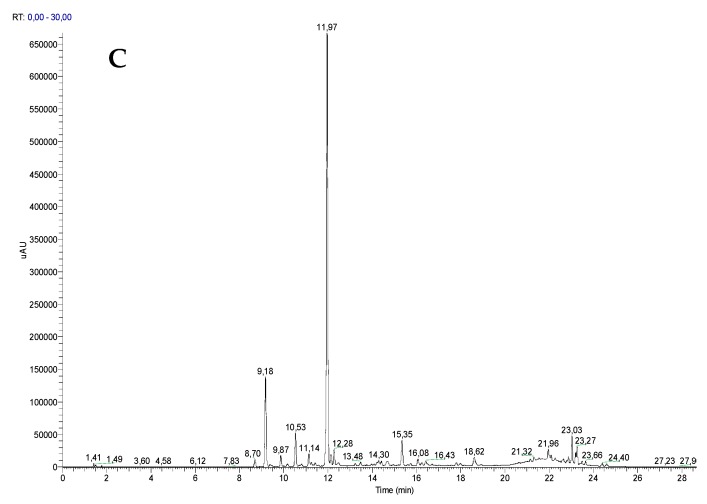
UHPLC chromatograms of *G. tridentata* extracts. (**A**) Stems extract (S) recorded at 230 nm; (**B**) leaves extract (L) recorded at 240 nm; (**C**) roots extract (R) recorded at 280 nm.

The analysis of the phenolic composition of *G. tridentata* three extracts was established using UHPLC-DAD-ESI/MS^n^ (ultra-high-performance chromatography coupled to photodiode-array detection and electrospray ionization/ion trap mass spectrometry) in the negative ionization mode. For each sample, three independent assays were carried out and similar intensities and nature were observed between the replicates. For each plant part extract the chromatograms were recorded at different wavelengths (Figure 6) but the compounds identification was performed for all samples at the same wavelength. The analysis of the plant parts extracts shows different profiles and that roots extract is less rich in phenolic compounds (Figure 6).

**Figure 7:**
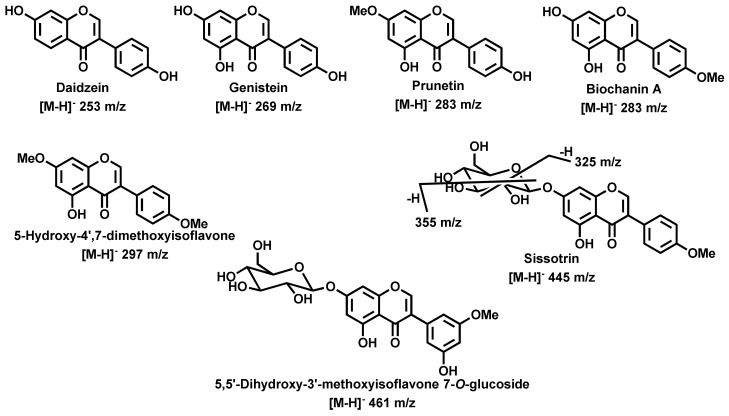
Isoflavonoid derivatives identified in *G. tridentata* extracts.

The compounds’ characterization was achieved by comparing the deprotonated molecular ion [M − H]^−^, although a few formate adducts ([M − H + CH_2_O_2_]^−^) were also observed, the fragments in MS^n^ and the UV spectra with literature data. Some available standards, both aglycones, and glycosides were injected in the same conditions to confirm some identifications. The analysis of the data resumed in Table 1, permits the confirmation that roots have fewer compounds, nine compounds were identified, whereas leaves and stems have a few extra derivatives. Among the identified compounds (Table 1), only genistein aglycone, one of its *C*-glycosides and 5,5′-dihydroxy-3′-methoxyisoflavone 7-*O*-glucoside (Figure 7) were found in the three analyzed extracts. On the other hand, several compounds were only found in one plant part extract (Table 1). These results also confirmed the richness of this species in isoflavone derivatives, as well as in glycosides. It should be emphasized that in our study the ethanolic extracts were analyzed, whereas in most reported studies water extracts were analyzed. Moreover, in this study the flowers were not included, which is the most studied plant part. These aspects explain the differences between the profiles herein discussed and the previously reported ones, in particular the previously reported richness in quercetin derivatives [8,20,33].

#### 2.2.1. Isoflavonoid Derivatives

Daidzein (Figure 7), only found in roots extract, exhibited [M − H]^−^ at *m*/*z* 253 and presented a fragment pattern consistent with literature available data [34], from which fragments at *m*/*z* 225 and 209, due to losses of CO and CO_2_, respectively, can be highlighted. Moreover, the isoflavones characteristic cleavages [34], that is fragments at *m*/*z* 135 and 117, respectively 1,3A^−^ and 1,3B^−^, can also be observed. Another important isoflavone found in all extracts was genistein (Figure 7). It exhibited [M − H]^−^ at *m*/*z* 269 and presented a fragment pattern (Table 1) consistent with literature available data [34], that allowed its assignment. The other isoflavone aglycones found were well known methylated derivatives of genistein (Table 1) that exhibited the expected molecular ions [M-H]^−^ and from which 5-hydroxy-4′,7-dimethoxyisoflavone (Figure 7), only found in stems extract and identified by other authors as methylbiochanin A/methylprunetin [8,20]. Biochanin A and prunetin (Figure 7) isomers can be easily distinguished by the fragment ions at *m*/*z* 165 and 151, respectively in prunetin and biochanin A, due to the 1,3A^−^ fragments. In the case of prunetin this fragment is consistent with the presence of a 7-OMe group (Figure 7).

The isoflavone glycosides found were also the expected ones, mainly genistein derivatives (Figure 7). Sissotrin, only found in stems extract, whose occurrence was previously reported in this species [8], presented the characteristic fragments at *m*/*z* 355, 325, and 283, due to losses of 90, 120, and 162 Da, characteristic when a glucoside moiety is present (Figure 7). Moreover, a fragment at m/z 267, indicating the loss of 178 Da, confirms the 7-*O*-glucose moiety. 5,5′-Dihydroxy-3′-methoxyisoflavone 7-*O*-glucoside (Figure 7), exhibited the expected molecular ion [M − H]^−^ and the observed fragmentation is similar to the previous reported mechanism [9].

Finally, the genistein glycosides presented the expected molecular ions [M − H]^−^ consistent with the presence of two or one glucoside moieties (Table 1). The derivative eluted at 9.15 min was assigned to a genistein diglucoside derivative, mainly due to its fragmentation consistency with losses of two glucose units. Furthermore, the loss of both 178 and 162 Da, corresponding, respectively, to *O*-glucose and glucose moieties allowed the assignment to a genistein *O*- and *C*-glucoside. However, in this study attempts to confirm the glucose moieties localization were not performed and we only know that it is a genistein *C*,*O*-glucoside derivative. A similar identification could be performed with the derivative eluted at 13.37 min, the occurrence of genistein 8-*C*-glucoside was previously reported [8,33], however we did not inject standards to confirm the localization of the glucose moiety. Two other isoflavone derivatives identified should be mentioned, the compounds eluted at 9.87 and 11.14 min, which were assigned to formic acid adducts. These adducts are common when a percentage of formic acid is used in the eluent, which was our case.

#### 2.2.2. Flavonol and Flavanonol Derivatives

**Figure 8:**
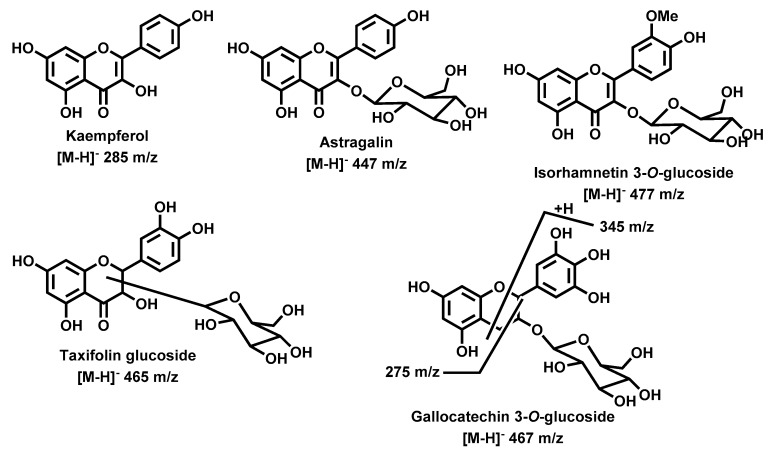
Other polyphenolic derivatives identified in *G. tridentata* extracts.

Kaempferol (Figure 8) was the only aglycone, from this type of flavonoids, found, all the others were detected in their glycoside form (Table 1). Interesting is the fact that both kaempferol and astragalin, the kaempferol 3-*O*-glucoside, were detected in stems extract. Apparently, this is the first report on their occurrence in this species, however their natural occurrence is well reported and our data are in accordance with the previously reported data, which were also used to establish our assignment [35,36,37,38].

Myricetin 6-*C*-glucoside (Figure 8), assigned to the compound eluted at 10.89 min, was previously found in the *G. tridentata* extract [14,33], as well as isorhamnetin 3-*O*-glucoside (Figure 8), assigned to the compound eluted at 10.89 min. [39]. In both cases our data are in accordance with the previous reported [14,33,39,40]. The presence of taxifolin glucosides in this species, in particular taxifolin 6-*C*-glucoside [8,33], was also reported. In this study taxifolin glucosides were found in all extracts, the compounds eluted at 8.59, 8.74, and 9.24 min (Table 1). Not only is our data similar to the previous reported one but also the occurrence of isomeric taxifolin glucosides is common [41], which explains our results. Although we detected three taxifolin glucosides we could not assign the exact position of the glucose moiety.

#### 2.2.3. Flavan-3-ol Derivatives

Compounds eluted at 9.76 and 10.64 min, were assigned respectively to gallocatechin 3-*O*-glucoside and epigallocatechin 3-*O*-glucoside (Figure 8) based on previous reported data concerning the flavan-3-ols fragmentation [41,42]. The losses of 162 and 178 Da, indicating the presence of an *O*-glucose unit, and the fragment ions *m*/*z* 345 and 275, common in flavan-3-ols (Figure 8), are the main characteristic of these compounds.

### 2.3. Characterization of the Isolated Metabolites

**Figure 9:**
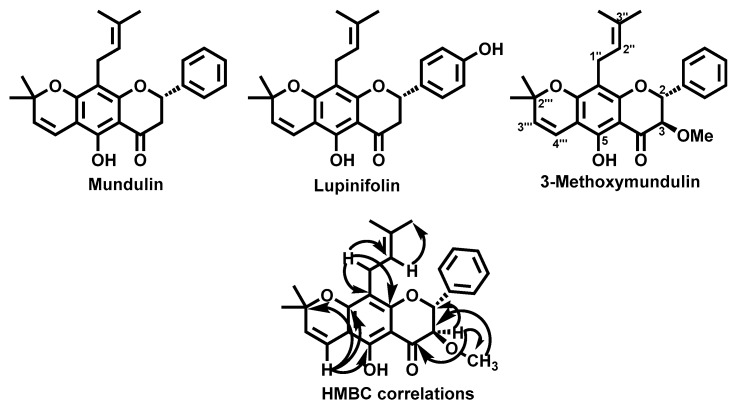
Prenylated flavonoids isolated from *G. tridentata* roots extract.

Due to the above-mentioned biological potential of the roots extract and the fact that this plant part was always neglected, indeed the plant aerial parts are the ones used in the medicines preparation [4], a phytochemical study was performed. Three prenylated flavonoids (Figure 9), lupinifolin, mundulin, and 3-methoxymundulin, a new natural compound, were isolated. First the extract was submitted to a liquid–solid extraction and a series of chromatographic techniques that allowed the purification of the mentioned flavanone derivatives. Comparison of the NMR spectroscopic data with the previous reported one led to the identification of lupinifolin [43] and mundulin [44], compounds found for the first time in this species. As far as we could find, 3-methoxymundulin (Figure 9) is a new natural derivative and consequently a careful characterization was performed. The mass spectrum showed peaks at *m*/*z* 421, 443, and 459, due to ions [M + H]^+^, [M + Na]^+^, and [M + K]^+^, respectively. The HRMS-ESI analysis showed a peak at *m*/*z* 421.2021 assigned to the molecular formula C_26_H_29_O_5_. The ^1^H-NMR spectrum indicated signals for a hydrogen-bonded hydroxyl group at δ_H_ 11.99 ppm (1 H, s, 5-OH), a monosubstituted aromatic ring at δ_H_ 7.44–7.48 ppm (5 H, m), two methyl groups at δ_H_ 1.43 (3 H, s, 2′″-CH_3_), and 1.45 ppm (3 H, s, 2′″-CH_3_), a methoxyl group at δ_H_ 3.39 ppm (3 H, s, 3-OCH_3_) and a vinylic system at δ_H_ 5.51 ppm (1 H, d, *J* = 9.2 Hz, H-3′″) and at δ_H_ 6.63 ppm (1 H, d, *J* = 9.2 Hz, H-4′″). The prenyl unit was identified from the resonance of one olefinic proton at δ_H_ 5.12 ppm (1 H, t, *J* = 6.0 Hz, H-2″), two methylene protons at δ_H_ 3.19 ppm (2 H, d, *J* = 6.0 Hz, H-1″) and two methyl groups at δ_H_ 1.64 ppm (6 H, s, 4″-, and 5″-CH_3_). Finally, the protons of the flavanone heterocyclic ring H-2 and H-3 resonate at δ_H_ 5.22 ppm (1 H, d, *J* = 10.0 Hz, H-2) and δ_H_ 4.04 ppm (1 H, t, *J* = 10.0 Hz, H-3), respectively. COSY correlations confirm olefinic protons assignments, as well as the assignments of both protons H-2 and H-3. The HMBC cross-peaks (Figure 9) joined with HSQC correlations allowed the carbons assignment, as well as the confirmation of the structure depicted in Figure 9.

Previous reports have revealed that both lupinifolin and mundulin present interesting biological activities, such as antimicrobial [45], antitumor [46], and ability to inhibit the H^+^,K^+^-ATPase enzyme [47]. Therefore, the isolation of this flavanone derivatives point out to the *G. tridentata* roots biological potential.

## 3. Materials and Methods

### 3.1. General Experimental Procedures

Solvents were purchased from Panreac and Acros Organics (Porto Salvo, Portugal) and were of HPLC purity, analytical grade, or bi-distilled commercial solvents. Chromatographic purifications were performed using silica gel 60 (Merck Kieselgel, 70–230 mesh) and Merck silica gel 60 GF_254_ (Oeiras, Portugal). Spots were detected in the TLC plates under UV light at λ 254 and 366 nm. The phenolic standards isorhamnetin, kaempferol, luteolin, apigenin, quercetin, and quercitrin were purchased from Extrasynthese (Genay Cedex, France) and were all standards for UHPLC that is 99% purity. The identification of individual phenolic compounds in the UHPLC analysis was achieved by comparison of their retention times, UV–Vis spectra and MS^n^ spectra data with those of the closest available reference standards and data reported in the literature.

The NMR spectra {^1^H, ^13^C, DEPT-135, DEPT-90, HSQC, HMBC [71 ms (7 Hz)], COSY} were measured in CDCl_3_, on a Bruker Avance 300 (300.13 MHz for ^1^H and 75.47 MHz for ^13^C) spectrometer and using TMS as internal standard. Chemical shifts were reported in δ units (ppm) and coupling constants (*J*) in Hz. The MS spectra were obtained using ESI^+^ with a Micromass Q-TOF2 mass spectrometer (Manchester, UK). The HRMS-ESI was obtained using a MicroTof spectrometer with Apollo II, using a voltage of 4500 V.

### 3.2. Plant Material

To conduct a representative analysis of the species, several specimens of the whole plant were collected during the flowering period (in August 2015) at “Serra da Estrela” region in Portugal and identified by the botanist Dr. Paulo Silveira, and a voucher specimen was stored in the Herbarium of the Department of Biology, University of Aveiro, Aveiro, Portugal (AVE6611). The plant specimens were dried and divided in roots, stems, and leaves. Each part was ground using a Retsch SKI equipped with a 40-mesh sieve.

### 3.3. Extract Preparation

The pulverized and air-dried parts of *G. tridentata* were extracted with ethanol using a Soxhlet (three cycles of 48 h). The plant part amount was put inside the cartridge and the ethanol was put in the round bottom balloon (10 g plant/250 mL solvent) and heated during 48 h. The solvent was removed and new ethanol was put in the balloon to make another extraction cycle. This extraction at the ethanol reflux temperature was used to mimic the infusion used in traditional medicine. The ethanol was evaporated under a rotary evaporator, yielding the respective extracts, which were used in the subsequent analyses. Roots extract (R): 83.6 g, 18% yield; leaves extract (L): 116.5 g, 30% yield; stems extract (S): 71.9 g, 20% yield.

### 3.4. Cell Culture

The murine macrophage cell line, RAW 264.7, was obtained from the American Type Culture Collection (ATCC: TIB-71) and grown in Dulbecco’s modified Eagle’s medium (DMEM) supplemented with 10% fetal bovine serum (FBS), 100 U/mL of penicillin, and 100 µg/mL of streptomycin. Cells were incubated at 37 °C in a humidified atmosphere of 95% of air and 5% of CO2 and were used after reaching 70%–80% confluence.

### 3.5. Cytotoxicity Assay

The effect of the different extracts on macrophage viability was assessed by the resazurin assay [48]. Briefly, 4 × 10^4^ cells were plated per well of a 96-well plate, left to stabilize overnight and then incubated with 500, 250, 100, 50, and 10 µg/mL of each extract during 23 h. The cells were then washed and a medium containing 50 µM of resazurin is added. The absorbance was measured at 570 and 600 nm in a BioTek Synergy HT spectrophotometer (Biotek Instruments, Winooski, VT, USA).

### 3.6. The Nitric Oxide Production and NO Scavenging Assays

Raw 264.7 cells were plated at 3 × 10^5^ cells/well in 48-well culture plates, allowed to stabilize for 12 h, and then treated with 1 ug/mL of LPS during 24 h. In certain conditions, cells were pretreated with 100 μg/mL of each extract. At the end of the incubation, 100 μL of culture supernatants were collected and mixed with an equal volume of Griess reagent [0.1% (*w*/*v*) *N*-(1-naphthyl)ethylenediamine dihydrochloride, and 1% (*w*/*v*) sulfanilamide containing 5% (*w*/*v*) H_3_PO_4_] during 30 min, in the dark. After 30 min incubation in the dark, the absorbance at 550 nm was measured using a BioTek Synergy HT spectrophotometer. Sodium nitrite (NaNO_2_) solutions (1–200 μM) were used to calculate a standard curve. The nitric oxide radical scavenging assay medium containing the NO donor *S*-nitroso-*N*-actyl-D/L-penicillamine (300 µM final concentration) and 100 µg/mL of each extract was incubated at 37 °C during 3 h. The level of NO was evaluated through the Griess reaction described above.

### 3.7. In Vitro Antioxidant Potential

Cells were plated at 0.05 × 10^6^ per well in a µ-Chamber slide (IBIDI GmbH, Germany), allowed to stabilize overnight and then treated with 1 µg/mL LPS during 16 h. When indicated, extracts (100 µg/mL) were added 1 h prior to LPS. At the end of incubation period, cells were washed three times and then loaded with 5 μM H_2_DCFDA and 0.5 μg mL^−1^ Hoechst in HBSS for 30 min at 37 °C in the dark. Cells were washed three times with HBSS and analyzed with an Axio Observer Z1 fluorescent microscope (Zeiss Group, Oberkochen, Germany) at 63× magnification.

### 3.8. The qPCR Analysis

Quantitative PCR (qPCR) was used to address the effect of extracts on the LPS-induced transcription of pro-inflammatory genes *Il1b*, *Nos2*, *Ptgs2*, *Il6,* and *Tnfa*. Total RNA was isolated with a TRIzol reagent according to the manufacturer’s instructions and the concentration determined by OD260 measurement using a NanoDrop spectrophotometer (Thermo Scientific, Wilmington, DE, USA). After reverse transcription, qPCR reactions were performed in duplicate for each sample on a Bio-Rad CFX Connect. Gene expression changes were analyzed using the built-in CFX Manager software using Hprt1 as a reference gene. This gene was experimentally determined with Genex software (MultiD Analyses AB, Göteberg, Sweden) as the most stable for the treatment conditions used. Primer sequences (Supplementary Table S1) were designed using Beacon Designer software version 7.7 (Premier Biosoft International, Palo Alto, CA, USA) and thoroughly tested.

### 3.9. The Western Blot Analysis

The capacity of *G. tridentata* extracts to mitigate LPS-triggered expression of the pro inflammatory proteins COX-2 and iNOS was evaluated by Western Blot. After treatments, cells were lysate in a RIPA buffer, protein concentration determined through BCA, and extracts denatured in a Laemmli buffer. Equivalent amounts of protein were applied and separated in SDS-PAGE gel 10% (*v*/*v*), being subsequently transferred to PVDF membranes (Amersham Biosciences, Uppsala, Sweden). The membranes were initially blocked with dry skimmed milk 5% (*m*/*v*) in Tris-buffered saline containing Tween-20 0.1% (*v*/*v*) (TBS-T), for 1 h at room temperature. Later, membranes were incubated overnight at 4 °C, with the primary rabbit antibodies to iNOS (1:10,000; R&D Systems, Minneapolis, MN, USA) and COX-2 (1:10,000; Abcam, Cambridge, UK). After washing in TBS-T, the membranes were incubated at room temperature for 1 h in secondary alkaline phosphatase-conjugated antibodies. Immunoreactive bands were visualized using ECF substrate on the FLA Thyphoon 9000 imaging system (GE Healthcare). To ensure loading of equal amounts of protein between conditions, membranes were stripped and reprobed with anti-tubulin antibody. The generated signals were analyzed using Total Lab 2009 software (TotalLab Ltd., Durham, NC, USA).

### 3.10. UHPLC-DAD-ESI/MS^n^ Analysis PCR Analysis

For the UHPLC-MS analysis, 25 mg of each extract were dissolved in 5 mL of methanol (final concentration 5 mg/mL) and the resulting solutions were filtered through a 0.2 µm nylon membrane (Whatman, Oeiras, Portugal). Three independent analyses were carried out for reproducibility. This technique was performed using a Thermo Scientific Ultimate 3000RSLC (Dionex) equipped with a Dionex UltiMate 3000 RS diode array detector and coupled to a mass spectrometer. The column used was a Thermo Scientific hypersil gold column (1000 × 20 mm) with a part size of 1.9 µm and its temperature was maintained at 30 °C. The mobile phase was composed of (A) acetonitrile and (B) 0.1% formic acid in water (*v*/*v*), both degassed and filtered before use. The flow rate was 0.2 mL/min. The elution gradient was 5% (solvent B) for 14 min, 40% (solvent B) over 2 min, 100% (solvent B) over 7 min, and the re-equilibration of the column with 5% of solvent B for 10 min. The injection volume was 2 µL. UV–Vis spectral data were gathered in a range of 190 to 450 nm and the chromatographic profiles were documented at 220, 230, 240, and 280 nm. The mass spectrometer used was an LTQ XL linear ion trap 2D equipped with an orthogonal electrospray ion source (ESI). The equipment was operated in a negative-ion mode with electrospray ionization source of 5.00 kV and ESI capillarity temperature of 275 °C. The full scan covered a mass range of 50 to 2000 *m*/*z*. Collision-induced dissociation MS/MS and MS^n^ experiments were simultaneously acquired for precursor ions. The detection and quantification limits (LOD and LOQ, respectively) were determined from the parameters of the calibration curves (Table S2).

### 3.11. Compounds Isolation and Purification

The roots extract (R), 53.53 g, was fractionated sequentially, at room temperature, with hexane, dichloromethane, ethyl acetate, acetone, and methanol. Each solvent was added to the extract and stirred for 2 h to dissolve some compounds, after it was evaporated and purified. From this liquid–solid extraction the hexane fraction (RH) and dichloromethane fraction (RD) were obtained. Therefore, the RH (1.86 g) was separated by column chromatography using a gradient solvent system of dichloromethane:acetone (100:0 to 0:100) and this yielded five subfractions: RH1 (464 mg), RH2 (645 mg), RH3 (160 mg), RH4 (195 mg), and RH5 (147 mg). Lupinifolin **1** (36 mg) was isolated from the subfraction RH5 by TLC and using dichloromethane as eluent. In addition, the RD (3.16 g) was separated by column chromatography using a gradient solvent system of dichloromethane:acetone:methanol (100:0:0 to 0:0:100) and this yielded thirty two subfractions which were combined according to their TLC profile. Mundulin **2** (5 mg) and 3-methoxymundulin **3** (3 mg) were isolated from the subfraction RD1 by TLC and using hexane:acetone (4:1) as eluent. The compounds NMR data can be found in the Supplementary Information.

### 3.12. Statistical Analysis

All the experiments were repeated three times or more, whenever the replicates were not statistically equal. The results are reported as mean values ± standard deviation of three independent experiments. Comparisons between two groups were made by the two-sided unpaired Student’s t test and multiple group comparisons by One-Way ANOVA analysis, with a Bonferroni’s Multiple Comparison or Dunnett’s test post-test. Statistical analysis was performed using GraphPad Prism, version 6 (GraphPad Software, San Diego, CA, USA). Significance levels are as follows: * *p* < 0.05, ** *p* < 0.01, *** *p* < 0.001, **** *p* < 0.0001.

## 4. Conclusions

**Figure 10:**
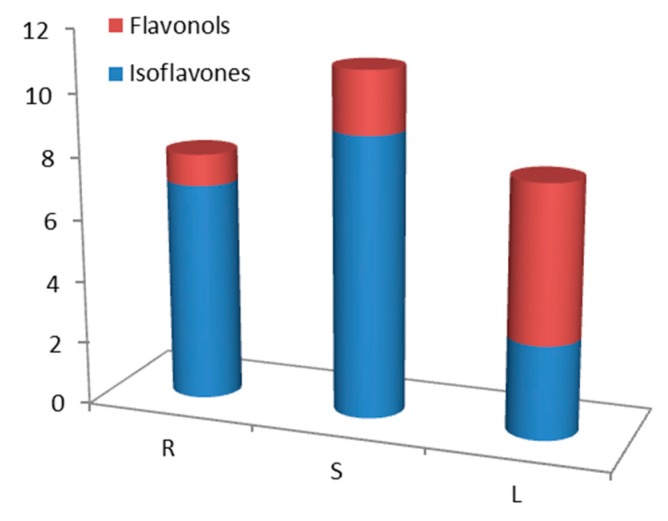
Representative types of flavonoids present in *G. tridentata* extracts.

Overall, the present study identified 20 polyphenolic compounds, among which 18 are flavonoids, in the ethanolic extracts of the roots, stems, and leaves of *G. tridentata*. Further investigation of fractions from root extracts led to the isolation of three prenylated flavonoids, including a newly described compound identified as 3-methoxymundulin. The biological assays demonstrated strong antioxidant and anti-inflammatory activities, supporting some of the plant traditional use in medicine. A brief analysis of the flavonoid type present in each extract (Table 1; Figure 10), shows that roots and stems are richer in isoflavones, whereas leaves have more flavonols, so it is expected that the anti-inflammatory activity observed is, in leaves due to the flavonols present, and in the other plant parts due to the isoflavones. It should be highlighted that both types present several hydroxyl groups in the flavonoid core, a characteristic associated with their anti-inflammatory activity.

Finally, our data indicate the roots of the shrub as an unexplored source of compounds with potential pharmacological and nutraceutical value. Considering that nutraceuticals are functional foods, that not only have nutritional value but also have some medicinal value [49,50], we believe that *G. tridentata* can be an ingredient of functional foods adding anti-inflammatory properties.

## Figures and Tables

**Table 1:** Flavonoid composition of *G. tridentata* ethanolic extracts. Retention time (t_R_; min.), wavelengths of maximum absorption in the visible region (λ_max_; nm), molecular ion ([M − H]^−^; *m*/*z*), mass spectral data (MS^n^; *m*/*z*), roots extract (R), leaves extract (L), and stems extract (S).

t_R_ *	λ_max_	[M − H]^−^	MS^2^	MS^3^	Assigned Identification	R	L	S
8.59	198, 224, 292, sh338	465	447(10) [M − H − H_2_O]^−^ 375(9) [M − H − C_3_H_6_O_3_]^−^ 345(100) [M − H − C_4_H_8_O_4_]^−^ 303(7) [M − H − glucose]^−^	357(9) [M − H − H_2_O − C_3_H_6_O_3_]^−^ 327(100) [M − H − H_2_O − C_4_H_8_O_4_]^−^ 285(11) [M − H − glucose − H_2_O]^−^ 195(11) [M − H − glucose − C_6_H_4_O_2_] 167(18) [M − H − glucose − C_7_H_4_O_3_]	^b,c^ Taxifolin glucoside I		✓	
8.69	192, 254, sh314	445	430(5) [M − H − CH_3_]^−^ 427(1) [M − H − H_2_O]^−^ 355(3) [M − H − C_3_H_6_O_3_]^−^ 325(7) [M − H − C_4_H_8_O_4_]^−^ 283(100) [M − H − glucose]^−^ 267(2) [M − H − Oglucose]^−^	268(100) [M − H − CH_3_ − glucose]^−^ 255(2) [M − H − glucose − CO]^−^	^c^ Sissotrin			✓
8.74	198, 229, 291, sh335	465	447 (10) [M − H − H_2_O]^−^ 375(10) [M − H − C_3_H_6_O_3_]^−^ 345(100) [M − H − C_4_H_8_O_4_]^−^ 303(7) [M − H − glucose]^−^	357(9) [M − H − H_2_O − C_3_H_6_O_3_]^−^ 327(100) [M − H − H_2_O − C_4_H_8_O_4_]^−^ 285(11) [M − H − glucose − H_2_O]^−^ 195(11) [M − H − glucose − C_6_H_4_O_2_] 167(18) [M − H − glucose − C_7_H_4_O_3_]	^b,c^ Taxifolin glucoside II		✓	
9.15	194, 260, 231, 330	593	575(10) [M − H − H_2_O]^−^ 503(7) [M − H − C_3_H_6_O_3_]^−^ 473(100) [M − H − C_4_H_8_O_4_]^−^ 431(7) [M − H − glucose]^−^ 415(2) [M − H − Oglucose]^−^ 269(7) [M − H − 2glucose]^−^	311(7) [M − H − glucose − C_4_H_8_O_4_]^−^ 325(2) [M − H − Oglucose − C_3_H_6_O_3_]^−^ 253(2) [M − H − Oglucose − glucose]^−^	^c^ Genistein *O*- and *C*-glucoside	✓		✓
9.24	199, 217, 290, sh336	465	447(80) [M − H − H_2_O]^−^ 375(15) [M − H − C_3_H_6_O_3_]^−^ 345(70) [M − H − C_4_H_8_O_4_]^−^ 303(30) [M − H − glucose]^−^ 285(100) [M − H − H_2_O − glucose]^−^	177(18) [M − H − H_2_O − glucose − C_6_H_4_O_2_]^−^ 151(15) [M − H − H_2_O − glucose − C_7_H_2_O_3_]^−^	^b,c^ Taxifolin glucoside III	✓	✓	
9.76	220, 265	467	449(10) [M − H − H_2_O]^−^ 345(100) [M − H − C_6_H_2_O_3_]^−^ 305(10) [M − H − glucose]^−^ 289(2) [M − H − Oglucose]^−^ 275(11) [M − H − CH_2_ − Oglucose]^−^		^b,c^ Gallocatechin 3-*O*-glucoside		✓	✓
9.87	194, 259, sh330	639	621(8) [M − H − H_2_O]^−^ 593(22) [M − H − CH_2_O_2_]^−^ 549(7) [M − H − C_3_H_6_O_3_]^−^ 519(83) [M − H − C_4_H_8_O_4_]^−^ 477(100) [M − H − glucose]^−^ 431(100) [M − H − CH_2_O_2_ − glucose]^−^	269(100) [M − H − CH_2_O_2_ − 2glucose]^−^ 549(22) [M − H − CO_2_ − CH_2_O_2_]^−^ 357(6) [M − H − glucose − C_4_H_8_O_4_]^−^ 315(7) [M − H − 2glucose]^−^	^d^ Formic acid genistein *O*- and *C*-glucoside adduct	✓		✓
10.49	198, 261, sh336	447	429(100) [M − H − H_2_O]^−^ 403(100) [M − H − CO_2_]^−^ 357(3) [M − H − C_3_H_6_O_3_]^−^ 327(6) [M − H − C_4_H_8_O_4_]^−^ 285(10) [M − H − glucose]^−^ 269(30) [M − H − Oglucose]^−^	267(23) [M − H − glucose − H_2_O]^−^ 257(7) [M − H − glucose − CO]^−^ 241(27) [M − H − glucose − CO_2_]^−^ 217(3) [M − H − glucose − C_3_O_2_]^−^ 177(11) ^0,4^B^−^ 149(16) [M − H − glucose − C_7_H_4_O_3_]^−^	^a,c^ Astragalin			✓
10.64	221, 265	467	449(10) [M − H − H_2_O]^−^ 345(100) [M − H − C_6_H_2_O_3_]^−^ 305(10) [M − H − glucose]^−^ 289(2) [M − H − Oglucose]^−^ 275(11) [M − H − CH_2_ − Oglucose]^−^	257(23) [M − H − H_2_O − CH_2_ − Oglucose]^−^	^b,c^ Epigallocatechin 3-*O*-glucoside		✓	✓
10.89	199, 220, 290, sh340	479	389(7) [M − H − C_3_H_6_O_3_]^−^ 359(100) [M − H − C_4_H_8_O_4_]^−^	341(36) [M − H − H_2_O − C_3_H_6_O_3_]^−^ 331(100) [M − H − CO − C_3_H_6_O_3_]^−^ 315(4) [M − H − CO_2_ − C_3_H_6_O_3_]^−^194(15) ^1,4^B^−^	^a,c^ Myricetin 6-*C*-glucoside		✓	
11.14	194, 255, sh328	491	445(100) [M − H − CH_2_O_2_]^−^ 283(24) [M − H − CH_2_O_2_ − glucose]^−^	283(16) [M − H − glucose]^−^ 268(100) [M − H − glucose − CH_3_]^−^ 255(1) [M − H − glucose − CO]^−^ 239(5) [M − H − glucose − CO_2_]^−^ 237(1) [M − H − H_2_O − CO]^−^	^d^ Formic acid biochanin *O*-glucoside adduct	✓		✓
13.37	206, 227, 249, 271, sh328	431	413(3) [M − H − H_2_O]^−^ 341(7) [M − H − C_3_H_6_O_3_]^−^ 311(100) [M − H − C_4_H_8_O_4_]^−^ 269(13) [M − H − glucose]^−^	293(15) [M − H − H_2_O − C_4_H_8_O_4_]^−^ 282(100) [M − H − HCO − C_4_H_8_O_4_]^−^ 225(4) [M − H − glucose − CO_2_]^−^	^c^ Genistein 6- or 8-*C*-glucoside	✓	✓	✓
13.69	192, 262, sh332	461	446(12) [M − H − CH_3_]^−^ 443(1) [M − H − H_2_O]^−^ 371(7) [M − H − C_3_H_6_O_3_]^−^ 341(7) [M − H − C_4_H_8_O_4_]^−^ 299(23) [M − H − glucose]^−^ 283(38) [M − H − Oglucose]^−^	326(3) [M − H − CH_3_ − C_4_H_8_O_4_]^−^ 268(2) [M − H − CH_3_ − Oglucose]^−^ 255(4) [M − H − CO − Oglucose]^−^	^c^ 5,5′-Dihydroxy-3′-methoxyisoflavone 7-*O*-glucoside	✓	✓	✓
14.73	196, 260, sh334	477	459(9) [M − H − H_2_O]^−^ 433(6) [M − H − CO_2_]^−^ 387(7) [M − H − C_3_H_6_O_3_]^−^ 357(70) [M − H − C_4_H_8_O_4_]^−^ 315(100) [M − H − glucose]^−^ 297(10) [M − H − H_2_O − glucose]^−^	297(75) [M − H − H_2_O − glucose]^−^ 282(100) [M − H − H_2_O − glucose − CH_3_]^−^ 269(13) [M − H − H_2_O − glucose − CO]^−^	^a,c^ Isorhamnetin 3-*O*-glucoside		✓	
15.35	255, sh318	283	268(100) [M − H − CH_3_]^−^ 255(2) [M − H − CO]^−^ 239(4) [M − H − CO_2_]^−^ 165(1) ^1,3^A^−^	196(1) [M − H − CH_3_ − CO_2_ − CO]^−^	^c^ Prunetin	✓		✓
16.08	249, sh295, 301	253	225(70) [M − H − CO]^−^ 211(3) [M − H − C_2_H_2_O]^−^ 209(7) [M − H − CO_2_]^−^ 135(2) ^1,3^A^−^ 117(1) ^1,3^B^−^	197(7) [M − H − CO_2_ − CO]^−^	^c^ Daidzein	✓		
16.57	198, 261, sh294	285	267(4) [M − H − H_2_O]^−^ 257(51) [M − H − CO]^−^ 241(27) [M − H − CO_2_]^−^ 217(53) [M − H − C_3_O_2_]^−^ 177(7) ^0,4^B^−^ 149(11) [M − H − C_7_H_4_O_3_]^−^	229(4) [M − H − 2CO]^−^ 199(72) [M − H − H_2_O − C_3_O_2_]^−^	^a,c^ Kaempferol			✓
17.09	194, 255, sh320	283	269(3) [M − CH_3_]^−^ 268(55) [M − H − CH_3_]^−^ 265(10) [M − H − H_2_O]^−^ 255(91) [M − H − CO]^−^ 151(1) ^1,3^A^−^ 132(1) ^1,3^B^−^ 149(4) ^0,3^B^−^	237(4) [M − H − H_2_O − CO]^−^ 212(5) [M − H − CH_3_ − 2CO]^−^ 181(3) [M − CH_3_ − 2CO_2_]^−^	^c^ Biochanin A		✓	
18.88	195, 261, sh332	269	251(12) [M − H − H_2_O]^−^ 241(86) [M − H − CO]^−^ 225(97) [M − H − CO_2_]^−^ 201(10) [M − H − C_3_O_2_]^−^ 151(10) ^1,3^A^−^ 117(4) ^1,3^B^−^	197(17) [M − H − CO_2_ − CO]^−^ 181(3) [M − H − 2CO_2_]^−^	^c^ Genistein	✓	✓	✓
21.03	194, 260, sh320	297	282(10) [M − H − CH_3_]^−^ 279(4) [M − H − H_2_O]^−^ 269(6) [M − H − CO]^−^ 253(3) [M − H − CO_2_]^−^	241(3) [M − H − 2CO]^−^ 225(5) [M − H − CO − CO_2_]^−^	^c^ 5-Hydroxy-4′,7-dimethoxyisoflavone			✓

* Average of three independent injections at the same wavelength (280 nm) ^a^ Identified based on MS^n^ and UV data, and comparison with a standard; ^b^ identified based on MS^n^ and UV data, and comparison with a similar standard; ^c^ identified based on MS^n^ and UV data, and comparison with other data from reference sources; ^d^ tentatively identified based on MS^n^ and UV data, and other literature evidence.

## Supplementary Materials

The following are available online: Table S1: Oligonucleotide primer pairs used for qPCR, Lupinifolin **1** NMR data, Mundulin **2** NMR data, 3-Methoxymundulin **3** NMR data.
