# Supplementary material for: Flavonoid Profile of the Genista tridentata L., a Species Used Traditionally to Treat Inflammatory Processes
Source: Molecules. 2020 Feb 13;25(4):812. doi: 10.3390/molecules25040812 (PMC7070917; doi:10.3390/molecules25040812)
Supplement: Supplementary file 1 [file molecules-25-00812-s001.pdf]

Flavonoid profile of *Genista tridentata* L., a species used traditionally to treat inflammatory processes

Mak A.M. Simões[a], Diana C. G. A. Pinto[a,*], Bruno M.R. Neves[b] and Artur M. S. Silva[a,*]

[a]LAQV-REQUIMTE, Department of Chemistry, University of Aveiro, Campus de Santiago, 3810-193 Aveiro, Portugal; [b]Department of Medical Sciences and Institute of Biomedicine – iBiMED, University of Aveiro, 3810-193 Aveiro, Portugal

Supplementary table 1: Oligonucleotide primer pairs used for qPCR.

Primer	5'-3' Sequence<br>F: Forward; R; Reverse	REFSEQ ID
Hprt1	F: GTTGAAGATATAATTGACACTG<br>R: GGCATATCCAACAACAAAC	NM_013556
Nos2	F: GCTGTTAGAGACACTTCTGAG<br>R: CACTTTGGTAGGATTTGACTTTG	NM_010927
Ptgs2	F: ATCAGACCTTCCTTGTAT<br>R: CACACTCATAGTTAAGACA	NM_011198.4
Il1b	F: ACCTGTCCTGTGTAATGAAAG<br>R: GCTTGTGCTCTGCTTGTG	NM_008361
Il6	F: TTCCATCCAGTTGCCTTC<br>R: TTCTCATTTCCACGATTTCC	NM_031168
Tnfa	F: CAAGGGACTAGCCAGGAG<br>R: TGCCTCTTCTGCCAGTTC	NM_013693

Supplementary table 2: Linearity (y = mx + b, where y corresponds to the standard peak area and x corresponds to the mass of standard), LOD and LOQ of pure compounds used as reference

Standard compound	Range concentration[§]	Slope (m)[§§]	Intercept (b)[§§]	R^2	LOD[§]	LOQ[§]
Isorhamnetin	0.5-100	629	-2316	0.9991	3	10
Kaempferol	0.5-175	792	-76	0.9969	5	17
Luteolin	0.5-100	354	-221	1.0000	3	10
Quercetin	0.5-175	317	-3	0.9992	4	13

[§]in µg/mL
[§§]in area counts/mg

Compounds NMR data:

Lupinifolin (**1**): orange oil; ^{1}H NMR (ppm, 300 MHz, $CDCl_3$): δ 1.44 (6H, s, H-5''' and H-6'''), 1.64 (3H, s, H-5''), 1.65 (3H, s, H-4''), 2.78 (1H, dd, *J* 17.1, 3.3, H-3eq), 3.04 (1H, dd, *J* 17.1, 12.8, H-3ax), 3.20 (2H, d, *J* 7.3, H-1''), 5.14 (1H, t, *J* 7.3, H-2''), 5.30-5.37 (1H, m, H-2), 5.50

(1H, d, *J* 10.0, H-3'''), 6.63 (1H, d, *J* 10.0, H-4'''), 6.87 (12H, d, *J* 8.5, H-3',5'), 7.31 (2H, d, *J* 8.5, H-2',6'), 12.23 (1H, s, 5-OH). RMN de ^{13}C (ppm, 75 MHz, $CDCl_3$): δ 17.8 (C-5''), 21.5 (C-1''), 25.8 (C-4''), 28.3 (C-5'''), 28.4 (C-6'''). 43.2 (C-3), 78.1 (C-2'''), 78.5 (C-2), 102.7 (C-8), 102.7 (C-10), 102.8 (C-6), 115.5 (C-3',5'), 115.7 (C-4'''), 122.5 (C-2''), 126.0 (C-3'''), 127.7 (C-2',6'), 130.9 (C-1'), 131.1 (C-3''), 156.0 (C-4'), 156.6 (C-5), 156.6 (C-9), 159.9 (C-7), 196.5 (C-4). MS ESI^+-TOF m/z: 407 $[M+H]^+$ (calcd for $C_{25}H_{26}O_5$ 406).

Mundulin (**2**): yellow oil; ^1H NMR (ppm, 300 MHz, $CDCl_3$): δ 1.44 (3H, s, H-6'''), 1.45 (3H, s, H-5'''), 1.66 (6H, s, H-4'' and H-5''), 2.70 (2H, dd, *J* 17.0, 3.0, H-3eq), 3.04 (1H, dd, *J* 17.0, 13.0, H-3ax), 3.22 (2H, d, *J* 7.4, H-1''), 5.16 (1H, t, *J* 7.4, H-2''), 5.23-5.47 (1H, m, H-2), 5.51 (1H, d, *J* 10.0, H-3'''), 6.64 (1H, d, *J* 10.0, H-4'''), 7.39-7.47 (5H, m, H-2',6'), 12.25 (1H, s, 5-OH). RMN de ^{13}C (ppm, 75 MHz, $CDCl_3$): δ 17.8 (C-5''), 21.5 (C-1''), 25.8 (C-4''), 28.3 (C-5'''), 28.4 (C-6'''), 43.5 (C-3), 78.1 (C-2'''), 78.8 (C-2), 102.7 (C-10), 102.9 (C-8), 108.6 (C-6), 115.7 (C-4'''), 122.5 (C-2''), 125.9 (C-3'''), 127.9 (C-2',6'), 128.6 (C-3',5'), 128.7 (C-4'), 131.1 (C-3''), 138.9 (C-1'), 156.6 (C-9), 159.2 (C-5), 159.9 (C-7), 196.2 (C-4). MS ESI^+-TOF *m/z*: 391 $[M+H]^+$, 413 $[M+Na]^+$, 429 $[M+K]^+$, (calcd for $C_{25}H_{26}O_4$ 390).

3-Methoxymundulin (**3**): yellow oil; ^1H NMR (ppm, 300 MHz, $CDCl_3$): δ 1.43 (3H, s, H-5'''), 1.45 (3H, s, H-6'''), 1.64 (6H, s, H-4'' and H-5''), 3.19 (2H, d, *J* 6.0, H-1''), 3.39 (3H, s, OCH_3), 4.04 (1H, d, *J* 10.0, H-3), 5.12 (1H, t, *J* 6.0, H-2''), 5.22 (1H, d, *J* 10.0, H-2), 5.51 (1H, d, *J* 9.2, H-3'''), 6.63 (1H, d, *J* 9.2, H-4'''), 7.44-7.48 (5H, m, H-2',3',4',5',6'), 11.99 (1H, s, 5-OH). RMN de ^{13}C (ppm, 75 MHz, $CDCl_3$): δ 17.8 (C-4''), 21.3 (C-1''), 25.8 (C-5''), 28.4 (C-5''' and C-6'''), 78.3 (C-2'''), 82.0 (C-2), 81.4 (C-3), 101.5 (C-10), 103.1 (C-6), 108.7 (C-8), 115.6 (C-4'''), 122.3 (C-2''), 126.1 (C-3'''), 127.1 (C-2',6'), 128.5 (C-4'), 128.9 (C-3',5'), 131.2 (C-3''), 136.8 (C-1'), 156.7 (C-5), 158.5 (C-9), 160.2 (C-7), 195.8 (C-4). MS ESI^+-TOF m/z: 421 $[M+H]^+$, 443 $[M+Na]^+$, 459 $[M+K]^+$. HRMS-ESI m/z 421.2021 $[M+H]^+$, (calcd for $C_{26}H_{29}O_5$ 421,2015).
